# High-resolution quantification of root dynamics in split-nutrient rhizoslides reveals rapid and strong proliferation of maize roots in response to local high nitrogen

**DOI:** 10.1093/jxb/erv307

**Published:** 2015-06-23

**Authors:** Dina in ‘t Zandt, Chantal Le Marié, Norbert Kirchgessner, Eric J.W. Visser, Andreas Hund

**Affiliations:** ^1^Department of Experimental Plant Ecology, Radboud University Nijmegen, Heijendaalseweg 135, 6525 AJ Nijmegen, The Netherlands; ^2^Crop Science, Swiss Federal Institute of Technology Zurich, Universitätsstrasse 2, 8092 Zurich, Switzerland

**Keywords:** Corn, foraging, nitrogen, rhizotrons, root growth, split-root.

## Abstract

The plant’s root system is highly plastic, and can respond to environmental stimuli such as high nitrogen (N) in patches. A root may respond to an N patch by selective placement of new lateral roots, and therewith increases root N uptake. This may be a desirable trait in breeding programmes, since it decreases NO_3_^-^ leaching and N_2_O emission. Roots of maize (*Zea mays* L.) were grown without N in split-nutrient rhizoslides. One side of the slides was exposed to high N after 15 d of root development, and root elongation was measured for another 15 d, described in a time course model and parameterized. The elongation rates of crown axile roots on the N-treated side of the plant followed a logistic increase to a maximum of 5.3cm d^-1^; 95% of the maximum were reached within 4 d. At the same time, on the untreated side, axile root elongation dropped linearly to 1.2cm d^-1^ within 6.4 d and stayed constant thereafter. Twice as many lateral roots were formed on the crown axis on the N side compared to the untreated side. Most strikingly, the elongation rates of laterals of the N side increased linearly with most of the roots reaching an asymptote ~8 d after start of the N treatment. By contrast, laterals on the side without N did not show any detectable elongation beyond the first day after their emergence. We conclude that split-nutrient rhizoslides have great potential to improve our knowledge about nitrogen responsiveness and selection for contrasting genotypes.

## Highlight

Patches rich in nitrogen are rapidly colonized by selective root growth in maize, which was quantified at high time resolution with state-of-the-art non-invasive imaging techniques in a paper-based growth system.

## Introduction

Sustainable agriculture is of utmost importance to feed the ever-increasing world population. Over the past few decades, higher agricultural yields have been achieved through an increase in, among others, fertilizer application, which increased 7.3-fold from 1960 to 2002 (FAO, 2014). However, agriculture is currently facing the challenge of reducing nitrogen (N) input due to legislative and public pressure in developed countries. Nitrogen fertilization causes pollution of ground, surface and coastal waters via NO_3_^-^ leaching (Galloway *et al.* 2003, 2004; ENA 2011) and increases N_2_O emission, which contributes to global warming (IPCC, 2013; UNEP, 2013). In this context, it is a crucial goal to decrease such N losses without reducing crop yield. A potentially interesting method to increase the fraction of N that is taken up by a crop is growing it under row fertilized conditions, which is the patch-wise application of fertilizer in the sowing row. An effective placement of roots would be needed to fully exploit the N patch supplied in this agrotechnique. However, studying root placement in the field in detail is difficult, due to neighbouring plants and the need for an invasive excavation of the root system. In this study, we therefore quantified the response of maize roots to a high N patch in a rhizoslide system (Le Marié *et al.*, 2014) that allowed high resolution measurements of root elongation and architecture over time.

Plants generally respond to a local high nutrient patch by increasing lateral root length specifically within the patch, which is referred to as selective root placement (Fransen *et al.*, 1998; Hodge, 2004; Visser *et al.*, 2008). Selective root placement is known to occur in a broad range of plant species (Robinson 1994; Cahill and McNickle 2011), including various cereals, in which increased lateral root elongation and lateral root density was observed in response to N patches (Hackett, 1972; Drew *et al.*, 1973; Drew, 1975; Granato and Raper, 1989; Yu *et al.*, 2014). Such an increase in root length and root density is important for nutrient uptake. Yet, the dynamics at which the roots respond to spatially variable nutrient supply is poorly understood. Hence, more detailed measurements are needed to determine which roots respond in which time frame to the locally increased nutrient concentrations. Zhang and Forde (2000) showed for *Arabidopsis* seedlings that lateral root elongation rate within the N patch increased linearly for the first 5 d to 3-fold of the original rate, while at the same time the elongation of the laterals outside the patch was only about half this rate and levelled off. This suggests a rapid re-allocation of growth towards the part of the root system directly in contact with the local high nutrient concentration. However, it remains doubtful if responses of seedlings can be easily translated to more complex stages of root architecture. This is particularly true for maize where the post-embryonic root system (crown roots) develops ~14 d after germination and plays a major role in nutrient and water uptake (McCully, 1999). A promising number of collocations were found between quantitative trait loci (QTLs) for the number of embryonic roots and traits related to grain yield (Hund *et al.*, 2011). However, only a few quantitative genetic studies focus on the characteristics of the crown root system (Hund *et al.*, 2011). The limited research conducted on the post-embryonic root system leaves a big gap in our understanding of the plasticity and functioning of this most prominent root type of maize. The embryonic root system is typically still strongly influenced by seed properties (Enns *et al.*, 2006; Hund *et al.*, 2009; Le Marié *et al.*, 2014), and growth and responses may not be representative for roots that develop later. Phenotyping systems, which allow the screening of a large number of plants at later stages of development, such as GROWSCREEN-Rhizo (Nagel *et al.*, 2012) and rhizoslides (Le Marié *et al.*, 2014), tackle this problem. There are different suggestions how the root system may optimize resource allocation to optimize the uptake of N and water. Lynch (2013) proposed an ideotype of maize to optimize water and N acquisition from deep soil layers. For this ideotype, the different root types specialize in different functions: embryonic roots are foraging for shallow soil resources, while crown roots expand rapidly deep into the soil, thereby being unresponsive to N availability. An alternative concept would be a strongly interactive framework of roots, of which the allocation towards different functions depends on the temporal and spatial availability of soil resources and the demand of the shoot. This would require nutrient sensing and coordinated growth regulation of the various components of the root system, as suggested by de Kroon *et al.* (2009). Indeed, in *Arabidopsis* low availability of N in parts of the root system is first signalled to the shoot which in turn triggers the expression of NO_3_^-^ transporter genes within root regions with high NO_3_^-^ content (Tabata *et al.*, 2014). Most likely, there will be an optimum, balancing between foraging of locally available N and the need to acquire a deep root system for sufficient water availability during grain filling. To evaluate the responsiveness of root growth on spatially varying N supply, methods are needed that enable measurement of the different root type elongation over time. Rhizoslides enable the evaluation of crown root development non-destructively and in detail by combining non-destructive regular imaging with subsequent image analysis (Le Marié *et al.*, 2014). Here, we present a further development of the rhizoslide system, enabling split-nutrient application. We aimed to test this system for its suitability to (i) observe crown root development in response to local N application in high temporal resolution, (ii) model and parameterize this response, and (iii) use the model parameters to evaluate the independence of growth of individual roots, growing on the same plant, when a couple of these roots were subject to an N patch. Moreover, this method allowed us to determine (iv) development of which root type was tightly linked to shoot growth and (v) the importance of the primary root on shoot and root development.

## Materials and methods

### Rhizoslide construction

Experiments were carried out in so called split-nutrient rhizoslides that were developed by Le Marié *et al.* (2014) and facilitate the two-dimensional growth of root systems over time alongside germination paper surfaces. The rhizoslides consisted of Plexiglas plates (length 65cm; width 57cm; thickness 4mm) with blue germination paper on each side (Anchor steel blue seed germination blotter; Anchor Paper Co., Saint Paul, Minnesota, USA; 61×48.4cm) covered with oxygen permeable transparent, polypropylene foil with micro holes of 70 µm (Maag GmbH, Iserlohn, Germany). The split-nutrient system was constructed by creating a water impermeable barrier from top to bottom in the germination paper by ironing into it a narrow band of wax (Wax pen, Knorr Prandell GmbH, Lichtenfels, Germany) (Supplementary Fig. S1A). After this, the germination paper was sterilized by heating in an oven at 80°C for 2h for three consecutive days (tyndallisation). During the time spans between the heating periods, the germination paper was kept in the oven at 30°C with a petri dish filled with deionized water to keep air humidity high (~50%). The germination paper was then impregnated with a 2.5g l^-1^ Captan solution (Malvin WG, Syngenta Agro AG, Dielsdorf, Switzerland) to suppress the development of fungi. Two PVC plates (60×10×1cm) were attached to the short side of the rhizoslides using flash washers as spacers between the Plexiglas plate and PVC plates. The PVC plates enabled the vertical placement of the rhizoslides in a rack protected against light. Two plants were grown in one rhizoslide, on each side of the Plexiglas plate one (Supplementary Fig. S1). Seedlings were placed in oval cylinders of black foil, filled with water-retaining material (Seramis clay granules, Seramis GmbH, Mogendorf, Germany) between the transparent foil and germination paper on top of the wax barrier (Supplementary Fig. S1B, C). To provide the plant shoots with sufficient space, the wax barrier was not placed exactly in the middle of the germination paper, but slightly (2.2cm) shifted to one side leaving 4.4cm room between the shoot of the plant growing on the front of the rhizoslide and the shoot of the plant growing on the back of the rhizoslide. This enabled the placement of seedlings on top of the wax barrier and at the same time next to each other. To prevent roots from being squashed, rubber flash washers were placed between the PVC plates and transparent foil on each side of the black foil cylinder (Supplementary Fig. S1B). The rhizoslides were each covered from the top with a flat PVC plate with two holes for the plants to grow through, to prevent incidence of light from above (Supplementary Fig. S1A).

The four compartments (two on each side of the Plexiglas plate) that were created by the wax barrier in the germination paper were watered separately. For this, four tubes were fixed at the top side of each compartment between the germination paper and the transparent foil.

The tubes were attached to drippers (Gardena pressure compensating row drippers supplemented with pumps and transformers from the Gardena Micro-drip Terrace irrigation system with additional timers; Gardena GmbH, Ulm, Germany) that were set to drip 35ml of nutrient solution twice a day. To prevent PO_4_^3-^ from precipitating, PO_4_^3-^ was kept together with the micronutrients and Fe-EDTA solution separate from the other nutrients and both solutions were mixed 30min before flush through (see ‘Plant cultivation’ for solution composition). To prevent roots suffering from hypoxia, the mixed nutrient solutions were bubbled with air prior to supplying them to the plants. To prevent fungal infections during plant growth, 0.066g l^-1^ of the fungicide Captan was added to the unmixed nutrient solutions (Malvin WG, Syngenta Agro AG, Dielsdorf, Switzerland). The pH of the unmixed nutrient solutions was adjusted to 6.0 with NaOH (final concentration Na^+^ ~20 µM).

### Plant cultivation

**Fig. 1.**
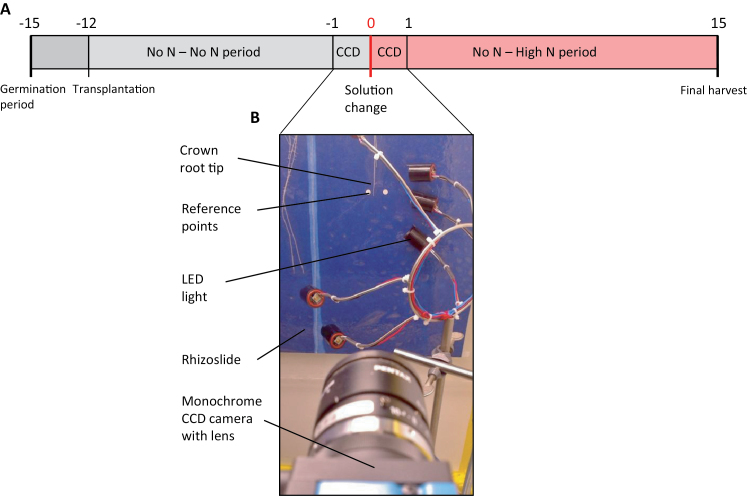
(A) Timeline of the experimental period with time indicated in days after solution change (DASC) and (B) setup of the Charge Coupled Device (CCD) measurements.

Maize seeds (*Zea mays* L., genotype B73×UH007, supplied by Delley Seeds and Plants Ltd, Delley, Switzerland) were sterilized in bleach (1.5% in water; v:v) for 15min and incubated in Petri dishes lined with filter paper soaked with a soil bacteria mixture (0.0001% RhizoPlus 42, Andermatt Biocontrol AG, Grossdietwil, Switzerland and 0.01% FZB24, Bayer AG CropScience, Zollikofen, Switzerland) to promote the development of a healthy rhizosphere and protect against seed-borne infections. During germination, seeds were kept in the dark at 26°C. After 70h, seedlings were transferred into the rhizoslides and placed in a climate chamber at 26°C during the 14h light and 20°C during the 8h dark period. Air humidity was 60% and light intensity 720−760 µmol m^-2^ s^-1^ photosynthetic photon flux density (PPFD) at plant canopy level supplied by 2/3 cool daylight tubes (Philips Master TL 5 HO 54W/840; Philips, Amsterdam, The Netherlands) supplemented with 1/3 Grolux tubes (Sylvania Grolux FHO 36W/T5/GRO; Havells India Ltd, Noida, India). Plants received nutrient solution without N containing 5mM KCl, 5mM CaCl_2_, 2mM MgSO_4_, 0.5mM KH_2_PO_4_, 0.04mM FeEDTA and micronutrients (9 µM MnCl_2_, 0.2 µM CuSO_4_, 46 µM H_3_BO_3_, 0.58 µM Na_2_MoO_4_ and 0.77 µM ZnSO_4_) in both compartments for 12 d after transplantation. The first 7 d after transplantation, plants also received nutrient solution without N twice a day at the shoot (40ml) to prevent the young roots from drying. The 12 d period without N was meant to minimize the influence of seed reserves and to create N deficient repression of selective root placement due to a high N content of the shoot. Twelve days after transplantation (set as time point 0; Fig. 1A), the nutrient solution in one compartment was changed to high N containing 5mM KNO_3_, 5mM Ca(NO_3_)_2_, 2mM MgSO_4_, 1mM NH_4_NO_3_, 0.5mM KH_2_PO_4_, 0.04mM FeEDTA and micronutrients (same as for the nutrient solution without N). Ion strength and free ion activity for both the solution with and without N were estimated using the chemical speciation programme GEOCHEM-EZ (Schaff *et al.*, 2010), and the solutions were adjusted accordingly to keep them as similar as possible with exception of the N concentration. The high N solution was used as a basis, and the nutrient concentrations in the high N solution were adjusted for which KNO_3_ and Ca(NO_3_)_2_ were replaced by KCl and CaCl_2_ (final concentration of Cl^-^ 15mM, which is considered non-toxic for maize; Ashraf, 1994) and NH_4_NO_3_ completely removed.

### Daily image capture and analysis

**Fig. 2.**
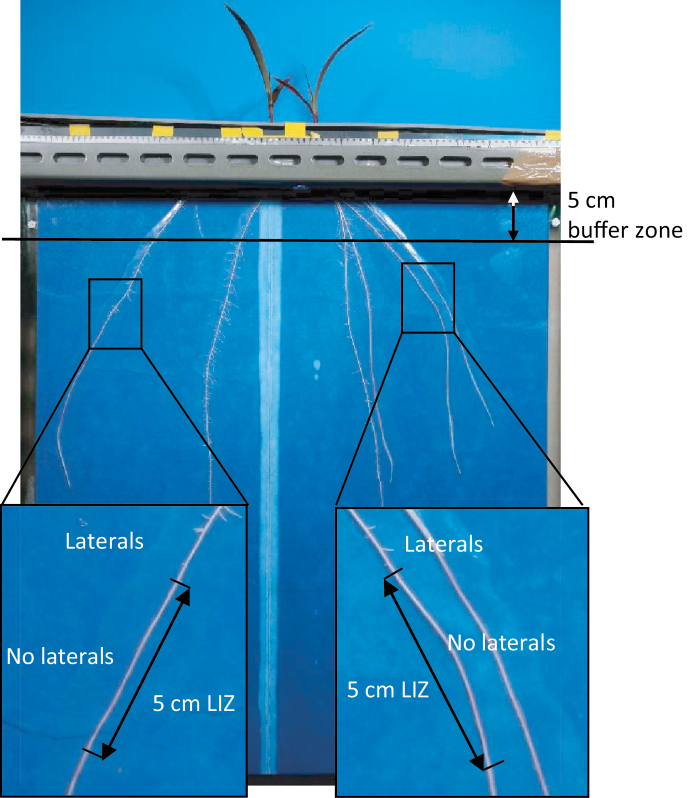
Selection of the lateral initiation zone (LIZ): after a 5cm buffer zone, the last initiated lateral was defined as the beginning of the analysed branching zone. The length of the LIZ was 5cm.

In each run, four plants were grown in the split-nutrient rhizoslide setup. From two of these, the primary root was cut off 2 d after transplantation into the rhizoslides. This was replicated five times over time resulting in 20 plants; 10 with and 10 without primary root. From 4 d after transplantation until the end of the growing period, daily images were taken of the whole root system with a 21 mega pixel full-frame digital single-lens reflex camera (EOS 5D Mark II, Canon, Tokyo, Japan) equipped with a 50mm lens (compact macro 50mm f/2.5, Canon, Tokyo, Japan) and circular polarizer (Hama, Augsburg, Germany). For this, plants were placed in a frame with the camera fixed at 125cm distance from the root system. Imaging and image pre-processing was carried out as described by Le Marié *et al.* (2014). As soon as a root reached the side or bottom of the germination paper, it was not traced any further. Tracing was stopped to avoid modelling of effects that were due to the size of the rhizoslide rather than the effect of the nutrient solution or root age. Crown roots reaching the bottom appeared not to influence elongation rates of associated laterals or other parts of the root system (data not shown). Next, root elongation of the crown roots over time was calculated using R (R Core Team, 2013). Crown roots that were not present at one day after solution change (DASC) were discarded, as well as roots that had less than four measurement points, and roots showing zigzag patterns or negative root elongation rates. In a few cases, roots were growing on the wax layer. In these cases, root elongation values were removed during these periods. Crown lateral roots were traced with SmartRoot after solution change (time point 0). For this a 5cm zone on the crown root axis was chosen, the lateral initiation zone, beneath the last formed laterals and taking a 5cm buffer zone from the PVC plate into account (Fig. 2). This lateral initiation zone could be determined at 1−2 DASC, and from then on, the number of laterals was determined each day. Additionally, the five laterals that initiated first in this zone, were traced with SmartRoot until the end of the experimental period. Root elongation was calculated for these laterals using R.

### CCD image capture and analysis

From -1 to 1 DASC, a crown root tip in the left compartment and a crown root tip in the right compartment of the rhizoslide of half the number of plants (one with and one without the primary root) was monitored with monochrome Charge Coupled Device (CCD) cameras (Scorpion SCOR-20SO, Point Grey Research Inc, Richmond, British Columbia, Canada) equipped with a 25mm lens (Cosmicar/Pentax, The Imaging Source, Bremen, Germany) and supplemented with a 940nm infrared filter (Edmund Optics, Karlsruhe, Germany). Plants were placed in a black box in which the roots to be monitored were illuminated with near-infrared LED lights (880/940nm) to avoid interference with root growth (Fig. 1B). Images were taken every 90sec at a resolution of 720×1280 pixels. After 22h (time point 0) the low N solution in one compartment was changed to high N solution. For this, the desired nutrient solution in both compartments was flushed through for 3min.

Images were analysed with a slightly adapted version of the Martrack Leaf software (Mielewczik *et al.*, 2013) that traced the root tip throughout the image sequences and a custom routine in Matlab calculated root elongation rates over time.

### Final harvest

After 30 d (15 DASC), plants were harvested (Fig. 1A). The shoot was cut off at the base and fresh weight was determined. After this, the shoot was bagged and dried in an oven at 60°C for at least 48h until weight stabilised, after which dry weight was determined. Roots were removed from the blue germination paper. The primary root, seminal roots from each compartment, crown roots from each compartment and roots growing within or on the other side of the germination paper were bagged and dried separately at 60°C for at least 48h, after which dry weight was determined.

### Statistical analyses

**Fig. 3.**
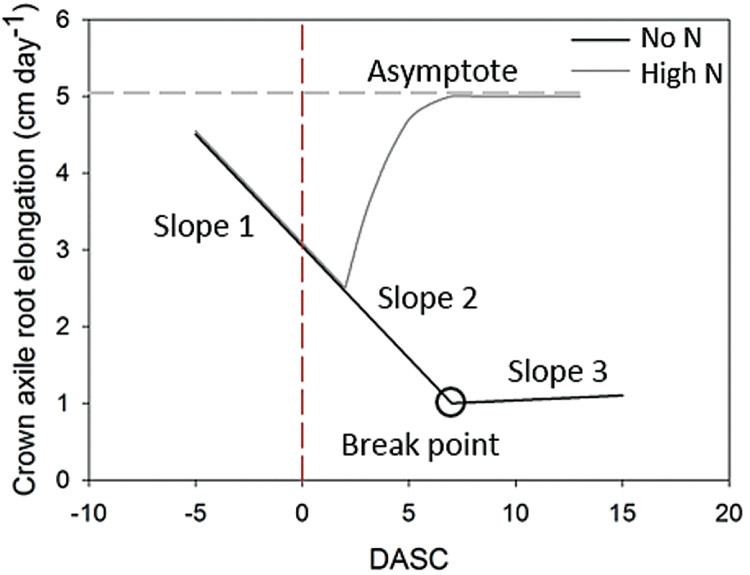
Root elongation parameters estimated from the models plotted for the no N and high N treated crown axile roots. The dashed, red line indicates the time point of solution change at 0 days after solution change (DASC).

The development of elongation rates of the different root traits was plotted using R and appropriate models were chosen according to the shape of the developmental curves (Supplementary Figs S2−S4). For crown root elongation before solution change, a simple linear regression was fitted (Fig. 3, Slope 1). For crown roots growing in the compartment without N after solution change, a regression model with segmented relationships and one breakpoint was fitted using the function *segmented*() of the R-package segmented (Muggeo, 2003, 2008). The estimated parameters were the slope after solution change (Fig. 3, Slope 2), the time at which the breakpoint occurred and the new slope after this breakpoint (Fig. 3, Slope 3). For the high N roots after solution change, a simple logistic model of the form

$$F\left(x\right)=\frac{\beta_{1}}{1+e^{-\left((\beta_{2}-x)/\beta_{3}\right)}}$$ Eq. 1

was fitted, were *ß*_*1*_ is the asymptote (Fig. 3), *ß*_*2*_ are the days after solution change at which half of the asymptotic value was reached, and *ß*_*3*_ is a scaling factor. Model fitting was done using the function *nls*() in combination with the self-starting function *SSlogis*() of the R-package stats (R Core Team, 2013). Spearman’s rank correlations were performed to determine the relationship among model parameter estimates and traits measured at final harvest. Significance of the differences per measured time point was tested with paired *t*-tests after Bonferroni correction between the no N and high N roots. Between plants, unpaired *t*-tests were performed to test for significant differences.

## Results

### Selective root placement

**Fig. 4.**
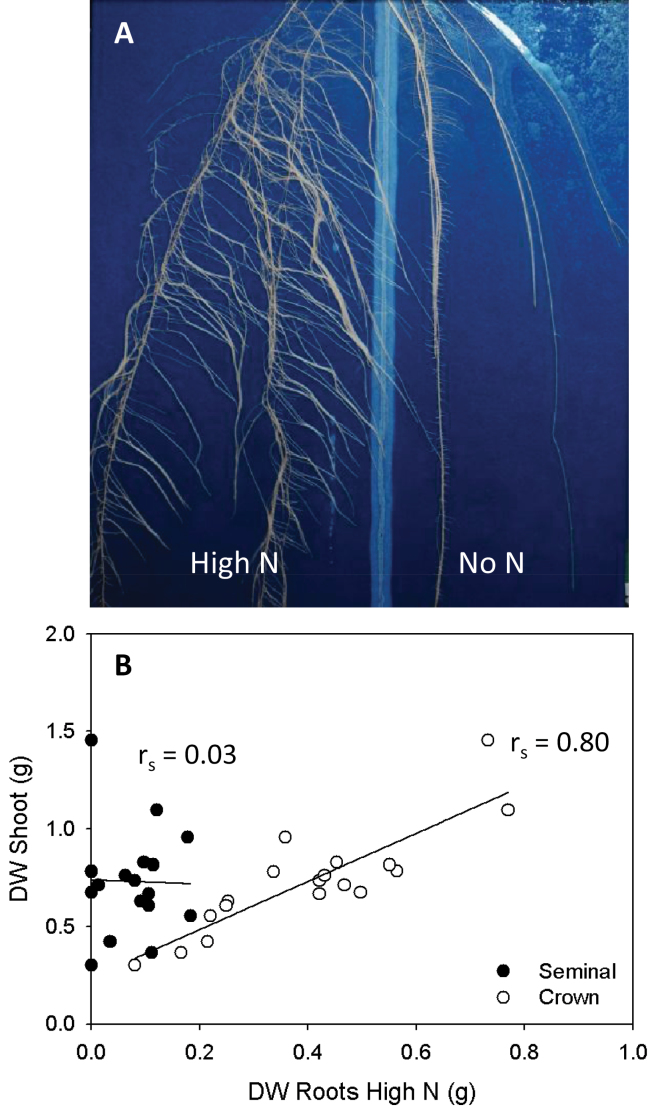
(A) Root distribution in a rhizoslide of 30-day-old *Zea mays* plants of which half the root system was subjected to high (17mM) N after 15 d of growing without N. (B) Correlations between dry weight (DW) of the shoot and DW of the seminal root system present in the high N compartment, and between DW of the shoot and DW of the crown root system present in the high N compartment. Significance tested with a Spearman’s rank correlation (r_s_; *n*=18).

To determine whether local application of N resulted in selective root placement, N was applied to only one half of the root system of 15-day-old maize seedlings previously grown without N. This resulted in a root biomass that was 3.6 times higher in the compartment that received N than in the compartment without N (Fig. 4A, Table 1). At the time of solution change, the crown root system had already started to develop while the seminal roots were 2 weeks old. Accordingly, the N placement had a strong effect on the developing crown roots which made up 85% of the selectively placed roots. The other 15% were represented by the seminal roots. The importance of the crown root system was also suggested by a positive correlation between dry weight of the part of the root system in the high N compartment and the dry weight of the shoot, whereas no correlation was found between biomass of the seminal root system in the same compartment and dry weight of the shoot (Fig. 4B). Based on these data, further analyses were done solely on the crown root system.

**Table 1.** Effects of local nitrogen application (NN, no nitrogen; HN, high nitrogen) on dry weight (DW) of the shoot and root of *Zea mays* plants

	DW Shoot (g)	DW Seminal (g)	DW Crown (g)
	0.730±0.06		
**NN**		0.032±0.01*	0.100±0.01***
**HN**		0.072±0.01	0.399±0.04

Plants were grown without N for 15 d after which half of the root system received 17mM nitrogen. Values are means ±SE (*n*=18). Asterisks indicate significance between LN and HN (paired *t*-test: ***, P<0.001; **, P<0.01; *, P<0.05; (*), P<0.1; NS, P<1.0).

### Crown root development

**Fig. 5.**
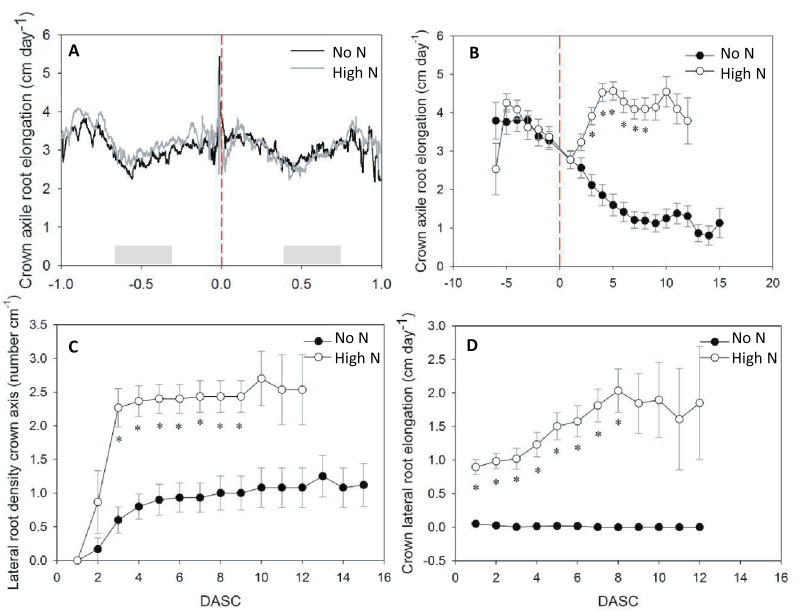
Root dynamics of crown axis and first order crown lateral roots in split-root rhizoslides subjected to either no nitrogen (N) or high (17mM) N after 15 d of growing without N. Crown axile root elongation determined by (A) tracking crown root tips with charge coupled device (CCD) cameras from -1 days after solution change (DASC) until 1 DASC, and (B) tracings of all the crown root tips over the whole experimental period. (C) Lateral root density on the crown root axis, and (D) elongation rates of these lateral roots. The dashed, red line indicates the time point of solution change, and the light grey squares night time. Values are means ±SE (*n*=2−13), asterisks indicate significant differences between the no N and high N roots (paired *t*-test with Bonferroni correction, *P*<0.05).

Individual crown root tip elongation was monitored in high time-resolution from one day before until one day after the onset of local high N supply (i.e. solution change), to investigate if selective root placement was an immediate response to increased N. We only analysed those six plants for which at least one root on the high N side and one root on the side without N could be tracked. Crown axile root elongation rates before solution change were on average ~3cm d^-1^ (Fig. 5A), with some diurnal variation (lowest rates in the afternoon, at 2.0cm d^-1^, and fastest rates in the late morning, at 4.0cm d^-1^). Crown axile roots that received high N showed a relative decrease of elongation rates in the first half hour after solution change, but recovered thereafter. Overall, crown axile roots that received high N did not show a clear change in elongation rate within the first day compared to roots of the same plant that did not receive N (Fig. 5A). Apparently, if crown axile roots respond to an increase in local N, this needs a longer initiation period than 1 d. Therefore, root elongation was monitored at longer time intervals during the following 15 d. In contrast to the first day, crown axile root elongation rates over the remainder of the experimental period showed a striking response to locally applied N (Fig. 5B, Table 2). The slightly slowing elongation rates just before solution change continued for roots kept without N until the breakpoint at ~6.4 DASC, after which root elongation rates more or less stabilized at a rate of 1.2cm d^-1^. Crown axile root elongation rates of roots supplied with high N, however, showed a dramatic increase in growth rates, levelling off to a maximum asymptote of on average 5.3cm d^-1^. To estimate the rapidness of this response, we calculated the time at which the roots reached 95% of their asymptotic value. This 95% of the maximum elongation rate was reached at approximately 4 DASC and for the majority of roots stayed at this maximum until the end of the experiment at 15 DASC (Fig. 5B, Table 2, Supplementary Fig. S2).

**Table 2.** Parameters describing crown axile and crown lateral root elongation over time of split-root no N (NN) and high N (HN) roots

	Crown							Lateral
	Slope (cm d^-2^)			Break point		Asymptote		Slope (cm d^-2^)
	1	2	3	x (DASC)	y (cm day^-1^)	x (DASC)	y (cm day^-1^)	
**NN**	-0.26 ^NS^	-0.41	0.02	6.4	1.2			0.00 ***
**HN**	-0.10					4.0	5.3	0.14

DASC, days after solution change. Crown axile and lateral root parameters were estimated from plotted models for Fig. 5B, with exception of the x-value of the asymptote, which was estimated directly from Fig. 5B, and crown lateral root initiation parameters, which were estimated from Fig. 5D. Values are means (*n*=6−13); asterisks indicate a significant difference between NN and HN roots (paired *t*-test: ***, P<0.001; **, P<0.01; *, P<0.05; (*), P<0.1; NS, P<1.0). See Fig. 3 for an explanation of the parameters.

### Lateral root development

A typical response of cereals to locally applied N is the development of more lateral roots within the N rich patch (Hackett, 1972; Drew *et al.*, 1973; Drew, 1975). To quantify this response over time, crown lateral root initiation was monitored after solution change, in both the part of the root system with high N and the part without N. Lateral root density on the crown root axis reached a maximum that was two to three times higher for the high N roots compared to the roots without N (1.0 laterals cm^-1^ without N, and 2.6 laterals cm^-1^ with N, respectively; Fig. 5C). Moreover, the time to reach this maximum differed considerably (4 d without N, and 2 d with N, respectively; Fig. 5C, Supplementary Fig. S3). Elongation rates of the initiated laterals were also influenced by the presence of N: without N, laterals initiated, but did not elongate beyond 1−5mm. Laterals in the compartment with N had already a higher elongation rate at 1 DASC, and showed every day a further increase in elongation rate of, on average, 0.14cm d^-1^ (Fig. 5D). This resulted in lengths of up to 18cm at 12 DASC, whereas the laterals without N only reached lengths of up to 1cm (Supplementary Fig. S5). The maximum asymptote for lateral root elongation for most laterals was reached at approximately 8 DASC, at on average 1.5−2cm d^-1^. A few roots, however, did not show a clear maximum asymptote within the experimental period (Supplementary Fig. S4).

### Correlation of root growth parameters with plant performance

We tested the correlation of the root dynamics during the different phases with final measurements at harvest. The stronger the elongation rate of crown axile roots declined before solution change (slope 1, Fig. 3A), the lower was the final root and shoot dry weight. Per gram of increase in dry weight of the total plant, slope 1 decreased with 0.20cm d^-1^ (Table 3). This indicates that the strong differences in dry weight between plants were established during the first 2 weeks of the experiment. After solution change, no significant correlations occurred anymore between biomass parameters and elongation rates of crown axile roots (data not shown) supporting the hypothesis that variation between plants was established early. After solution change, the time point at which 50% of all crown laterals in the high N compartment were formed, was positively correlated with dry weight of the shoot. A 0.1g increase in dry weight of the shoot was associated with an almost half day earlier formation of 50% of all crown laterals in the high N compartment (Table 3). Thereafter, the acceleration of elongation of these laterals in the high N compartment was negatively correlated with dry weight of the roots in the same compartment (seminal plus crown root system; Table 3). With each gram of roots that was more present in the high N compartment, the slope of lateral root elongation decreased with 0.6cm d^-1^.

**Table 3.** Correlations between parameters describing root development in a split-root system with half the root system supplied with no N (NN) and the other half with high N (HN)

Parameter 1	Parameter 2	r_s_	Significance
Slope 1	DW total plant	-0.76	*
Slope 1	DW roots	-0.67	*
Slope 1	DW shoot	-0.60	(*)
Slope 1	FW shoot	-0.71	*
Reached 50% HN	DW shoot	0.89	*
Slope lat HN	DW roots HN	-0.89	*

DW, dry weight; FW, fresh weight; ‘Reached 50% HN’, time point at which 50% of all laterals were formed in the high N compartment; ‘Slope lat HN’, acceleration of elongation of laterals in the high N compartment. If not indicated otherwise, no N and high N root parameters were taken together. Spearman’s rank correlation coefficient (r_s_) indicates statistical dependence (*n*=6−9; (paired *t*-test: ***, P<0.001; **, P<0.01; *, P<0.05; (*), P<0.1; NS, P<1.0). See Fig. 3 for model estimated parameters.

### Effect of primary root removal

In split-root experiments, the primary root may cause a bias, since it can only be placed in one of the compartments and thus affect root biomass in that compartment. In this study, the primary root of half of the plants was removed 2 d after transplantation to determine the influence on selective root placement. Significant effects occurred neither on the biomass of the shoot and total root system nor on the dry weight of the crown root system (Supplementary Fig. S6).

A significant 2-fold increase in dry weight of the total seminal root system was found when the primary root was removed (Supplementary Fig. S6). However, when seminal root biomass in the separate compartments without and with high N was compared between plants with and without primary root, this difference disappeared (data not shown). The model-derived parameters, as well as crown root emergence, were not significantly affected by primary root removal. A trend indicated, however, a 2-fold higher elongation rate from 1.0 to 2.0cm d^-1^ at the breakpoint for crown roots grown without N, when the primary root was removed (data not shown). Despite the comparably high number of replications, insufficient data were available to proof the significance of the primary root placement in either the low or high N compartment for the growth dynamics of the remaining root system.

## Discussion

### N patches cause faster elongation of crown roots

The elongation of crown roots increased asymptotically upon exposure to high N to an average of 5.3cm d^-1^ at ~4 DASC. It is well established, that a heterogeneous distribution of N in the soil can lead to selective root placement resulting in a higher root length in the N patch than in the surrounding soil (e.g. Hackett, 1972; Drew, 1975; Fransen *et al.*, 1998; Zhang and Forde 1998; Remans *et al.*, 2006). This response could, however, only be observed on the slightly long term, i.e. from daily images, and not on the short term, i.e. on the high-time-resolution CCD measurements. The CCD measurements did not reveal a short-term response by changes in root growth, whereas root growth started to increase 24−48h after solution change. This timing is in line with observations of Tabata *et al.* (2014) where NO_3_^-^ transporters were up-regulated ~24h after exposure of Arabidopsis roots to N-rich medium. Similar dramatic increases in elongation rates were not observed in seminal roots in experiments by Drew *et al.* (1973) on barley. These roots grew from a low NO_3_^-^ compartment into a high NO_3_^-^ compartment, and showed only a slight increase 17.5 d after entering the high NO_3_^-^ compartment. This difference in response might be related to the internal N status of the shoot. Our maize plants were likely to be N starved from growing without N for 12 d, while the roots of the barley plants in the experiments of Drew *et al.* (1973), with exception of one seminal root, were growing on high NO_3_^-^, causing a higher internal N status, and therefore possibly a slower and less prominent response. This hypothesis is quite likely, as N starvation on one side of the root system, leading to an up-regulation of NO_3_^-^ uptake on the other side of the root system, is mediated by the shoot (Tabata *et al.*, 2014). The reason for this indirect pathway may be a check for the N status of the shoot. We found that root elongation on the side without N declined until a ‘change point’ of 1.2cm d^-1^ ~6.3 d after solution change. This decrease is consistent with the decrease in elongation rates of the seminal root axes of barley growing from a low NO_3_^-^ compartment into another low NO_3_^-^ compartment (Drew *et al.*, 1973). Also here, a breakpoint was suggested for the elongation rate of these roots. Possibly, allocation of resources from the shoot to these roots in N-deficient conditions was gradually declining.

### N patches strongly stimulate lateral root production with most dramatic effects on lateral root elongation

The total lateral root length increased within the high N patch, which resulted from both longer lateral roots and an increase in lateral root density on the crown root axis. Selective root placement has most often been reported as an increase in lateral root production (e.g. Hackett, 1972; Drew *et al.*, 1973; Fransen *et al.*, 1998; Zhang and Forde 1998; Remans *et al.*, 2006). The increase of both length and densities of lateral roots is consistent with findings for barley (Drew *et al.*, 1973; Drew, 1975), wheat (Hackett, 1972) and maize (Granato and Raper, 1989), but is in contrast with findings for *Arabidopsis* where only lateral root length increased and not lateral root density (Zhang and Forde, 1998; Zhang *et al.*, 1999). However, *Arabidopsis*, in contrast to the cereals barley, wheat and maize, only has laterals developing from the primary root, and therefore an entirely different type of root architecture. For maize it has, however, also been suggested that a local high NO_3_^-^ concentration does not affect lateral root density on the primary root axis (Yu *et al.*, 2014). This divergent observation might be caused by a difference in developmental stage, since very young seedlings were used in the latter study (7 d after germination), with a strong influence of seed reserves on root morphology as demonstrated by Enns *et al.* (2006).

The lateral root density in the 5cm observation zone increased to a maximum within 3−4 d. The final density under high N was with 2.3 roots cm^-1^ about two to three times higher compared to the density at the side without N. These values are still lower than those found in solution and sand culture for maize (Yu *et al.*, 2014). One explanation could be the delayed formation of new lateral roots. To verify this hypothesis, we counted lateral roots in the second half of the rhizoslide and found lateral root densities of 4.5 laterals per cm on the high N side and 1.1 laterals per cm on the side without N. However, the branching density observed by Yu *et al.* (2014) was still higher. This could probably be a result of the use of a different genotype, and cultivation systems with more contact between roots and substrate. In rhizoslides, only about half of the root is in contact with the paper substrate while the other half is exposed to the foil cover, which may limit development of laterals. Hund *et al.* (2009) however used a similar system and found ~7 lateral roots per cm primary axile root for most extreme genotypes. Since primordia numbers were not determined in the present study, it is unclear whether this was resulting from a higher percentage of primordia emergences or from the presence of more primordia. However, Jordan *et al.* (1993) suggested that maize typically lacks dormant root primordia, favouring the second hypothesis. In the compartment without N, it took twice as long to reach the maximum lateral root density of the first order laterals on the crown axis compared to in the high N compartment. After reaching this maximum, no additional laterals were formed on that particular part of the crown root axis in both the compartments with and without N. This suggests that once first order laterals were formed, no additional laterals could be produced in response to the prevailing conditions, as also found for wheat (Hackett, 1972). Carbohydrate allocation may play an important role in the increase in lateral root densities on the root axis, as suggested by the increase in lateral root density in both *Arabidopsis* and wheat in response to glucose or sucrose feeding (Bingham *et al.*, 1998; Crookshanks *et al.*, 1998). Tobacco roots, however, did not show a similar response (Stitt and Feil, 1999). To date it is unknown if these effects were results of previous carbon limitation or if the sugars acted as signal molecules (Forde, 2002).

The most remarkable change in the whole root system was the prolonged elongation of the lateral roots exposed to high N, while those without N stayed short. The elongation rates for most laterals under high N in our experiment increased linearly to values as high as 2.0cm d^-1^. Some growth rates even proceeded to increase linearly until the end of the experimental period (15 DASC; Supplementary Fig. S4). This is clearly not in line with previous work, where first order lateral roots of crown and seminal roots of maize were short, reaching only ~3cm in length (McCully, 1999; Hund *et al.*, 2007, 2011) and only ~10% of the laterals reach a length greater than 10cm (Pagès and Pellerin, 1994). Usually an exponential decrease in elongation rates is assumed for first order laterals of maize (Pagès *et al.*, 1989). Prolonged elongation rates are usually only known for the primary lateral root of some genotypes of maize (Hund *et al.,* 2004, 2007). It seems that the response to a spatially varying N concentration allows the root system to respond with prolonged and fast elongation of crown lateral roots.

Compared to a study on barley (Drew *et al.*, 1973), we observed an elongation rate that was more than three times greater. An important factor in this may have been, again, the stronger N deficiency of the maize plants in our study.

### Removal of the primary root affects growth of the other root types

The position of the singular primary root in a split-root setup, being either in the compartment with or without N, could potentially affect the response of other parts of the root system. This is why the primary root is often partially or completely removed at the start of the experiment (e.g. Granato and Raper, 1989; Schortemeyer *et al.*, 1993; Marschner and Baumann, 2003; Shen *et al.*, 2005; Remans *et al.*, 2006). Such removal of the primary root in our experiments had a significant positive effect on the size of the seminal root system, suggesting an early competition between primary and seminal roots for carbon. However, the final total size of the crown root system was not significantly influenced. Still, a 2-fold higher elongation rate of the crown roots without N at the breakpoint of the segmented regression was apparent for plants without their primary root. Removal of the primary root thus influenced the seminal and, to a lesser extent, the crown root system. A thorough consideration of how to cope with primary root position in split-root setups is therefore important, especially when studying the embryonic root system.

### No direct dependence of the dynamic of roots exposed to differential N supply

The two to three times greater lateral root density on the crown root axis, the striking increase in lateral root elongation, and the fast increase in crown axile root elongation clearly indicate that the plants prioritized N uptake from the N patch. On the other hand, only few laterals were formed in the compartment without N, which ceased to elongate within the first day after formation. We correlated the estimated model parameters of the different root-response models to evaluate their dependency. There was no correlation between these model parameters determined under no N conditions and those determined under high N. For example, the time until 95% of the asymptotic value was reached for crown root elongation on the high N side was not correlated with the time until the break point was reached on the no N side. This indicates a large degree of independency. In the case of a direct dependency within the network of roots, we would have expected a correlation among different parameters. The lack of direct dependencies may be plausible when considering the shoot as an organ integrating signals from roots without N to roots with high N (Tabata *et al.*, 2014). We measured shoot traits only at the end of the experiment. Nevertheless, the correlation between the early decline in root elongation rates and plant weight indicates that differences among plants established before solution change. After solution change, later larger plants seemed to be able to form lateral roots in response to N more rapidly. Clearly, a similar dynamic for the shoot would benefit the interpretation of these root-shoot-root interactions. For this reason, we added shoot imaging to the next version of the rhizoslide system.

### Perspectives for application

The strong selective root placement demonstrates the exceptional plasticity of the maize crown roots, and the strong ability of the B73×UH007 hybrid to respond to local N patches. The mother inbred line B73 is one of the most successful inbred lines, being incorporated in many breeding programmes throughout the world. It may be speculated that the strong responsiveness of crown roots to N is part of its success, which would somehow contradict the philosophy to only select for unresponsive root systems, as proposed by Lynch (2013). However, we think that the extreme responses observed here were likely caused by severe N-starvation in the first 2 weeks of growth. In a follow-up study, with 1.7mM N during the first 2 weeks, the response was more moderate and the length of the lateral roots was ~3cm as observed usually (data not shown). Most plants will seek for an optimization of N uptake to maintain organ growth depending on their nutrient status. Such foraging behaviour would support the concept that also a generally high responsiveness to locally varying nutrient supply could benefit the plant if coping with heterogeneous nutrient conditions. A modelling study by Dunbabin *et al.* (2004) showed that a root system capable of strong selective root placement to N displayed a >2-fold higher N uptake efficiency throughout the whole growing season, compared to a root system that was only poorly capable of selective root placement to N. This suggests that particularly when N fertilizer is applied side-banded during sowing, varieties with the ability of a strong selective root placement may benefit most and, subsequently, decrease NO_3_^-^ leaching (Dunbabin *et al.*, 2003). Maize seems to be a suitable species to start such experimental systems, since it has been shown that it is capable of finding and colonizing a fertilized furrow in the field (Skinner *et al.*, 1998; Chassot *et al.*, 2001). Great potential thus seems to exist for improved N management through patch-wise N fertilization in such a cropping system.

Only a few quantitative genetic studies focus on the characteristics of the crown root system (Hund *et al.*, 2011) and the limited research conducted on the post-embryonic root system leaves a big gap in our understanding of the plasticity and functioning of this most prominent root type of maize. The rhizoslide setup combined with the root growth modelling approach presented could be a first step in the direction of high-throughput screening allowing detection of QTLs responsible for favourable root traits under limited N; whereas the most time-intensive step is still the image analysis. As the lateral root tracing is time-intense, we would suggest counting branching densities and determination of lateral root length only at critical time points. For example, this study demonstrated that the last time point would be representative of branching density. To screen crown roots of a population of 1000 individual plants in the manner proposed here, the image analysis would entail around one month’s work, a feasible option in our opinion.

## Supplementary data

Supplementary data is available at *JXB* online.

Supplementary Figure S1. Rhizoslide construction.

Supplementary Figure S2. Root elongation rates of individual maize crown roots grown in a split-root rhizoslide system.

Supplementary Figure S3. Lateral root density on the crown root axis of maize for roots grown in a split-root rhizoslide system.

Supplementary Figure S4. Lateral root elongation rates of individual roots of maize of crown roots grown in a split-root rhizoslide system.

Supplementary Figure S5. Crown lateral root length of maize plants over time grown in a split-root rhizoslide system with half the root system in no N and the other half in high N.

Supplementary Figure S6. Dry weight (DW) of the seminal and crown root system of maize plants grown in rhizoslides.

Supplementary Data
