# Supplementary material for: High-resolution quantification of root dynamics in split-nutrient rhizoslides reveals rapid and strong proliferation of maize roots in response to local high nitrogen
Source: J Exp Bot. 2015 Jun 23;66(18):5507–17. doi: 10.1093/jxb/erv307 (PMC4585423; doi:10.1093/jxb/erv307)
Supplement: Supplementary Data [file supp_66_18_5507__index.html]

High-resolution quantification of root dynamics in split-nutrient rhizoslides reveals rapid and strong proliferation of maize roots in response to local high nitrogen — High-resolution quantification of root dynamics in split-nutrient rhizoslides reveals rapid and strong proliferation of maize roots in response to local high nitrogen — Supplementary Data 

# High-resolution quantification of root dynamics in split-nutrient rhizoslides reveals rapid and strong proliferation of maize roots in response to local high nitrogen

## Supplementary Data

Data files

- Supplementary Data - Supplementary Data
