# Supplementary material for: High-resolution quantification of root dynamics in split-nutrient rhizoslides reveals rapid and strong proliferation of maize roots in response to local high nitrogen
Source: J Exp Bot. 2015 Jun 23;66(18):5507–17. doi: 10.1093/jxb/erv307 (PMC4585423; doi:10.1093/jxb/erv307)
Supplement: Supplementary Data [file supp_erv307_jexbot149765_file001.pdf]

Supplementary Figure 1

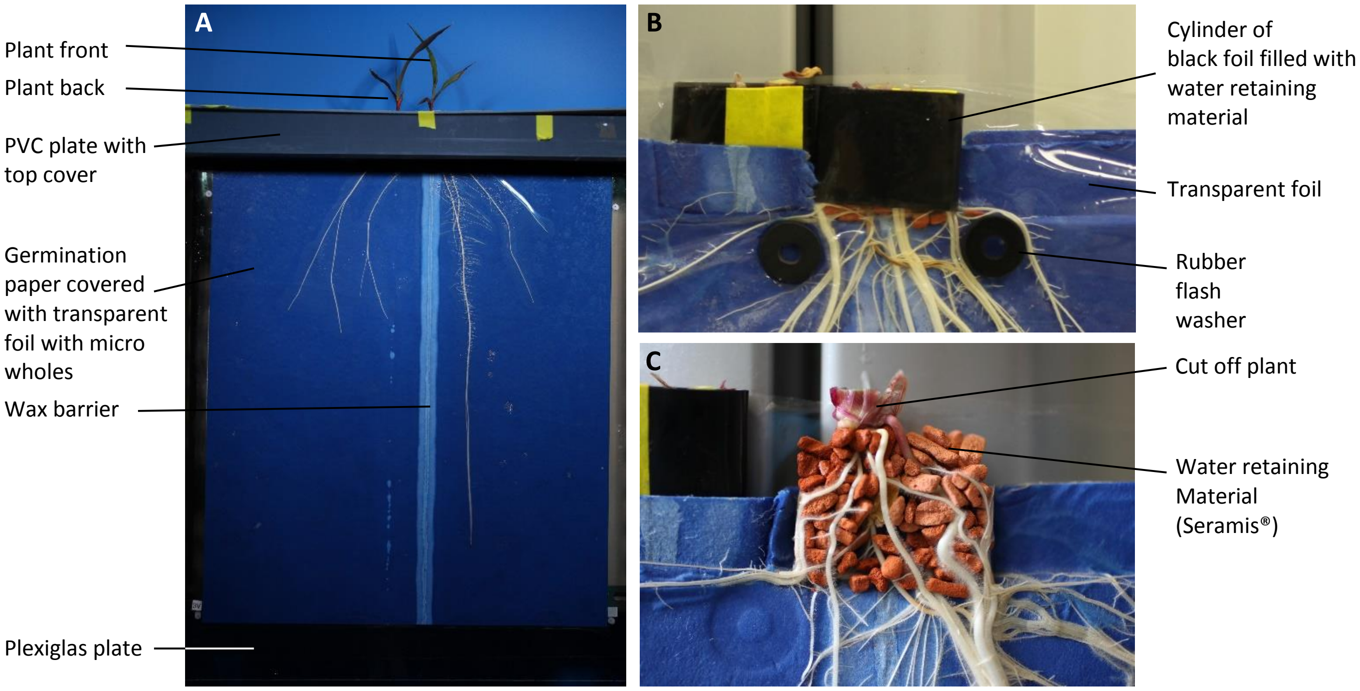

Supplementary Figure 1 (A) The split-root rhizoslide system, (B) top of the rhizoslide without the PVC plates and top cover, and (C) with the transparent and black foil cylinder removed.

Supplementary figure 2

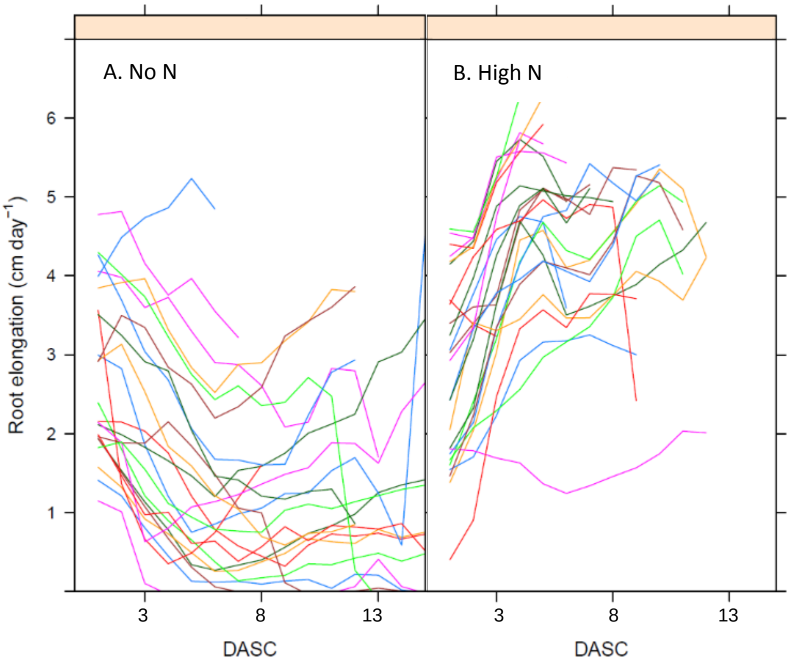

Supplementary Figure 2 Root elongation rates of individual maize (*Zea mays* L.) crown roots grown in a split-root rhizoslide system with half the roots in a no N compartment (A) and the other half in a high N compartment (B) over time. Different coloured lines indicate different roots and time is given in days after solution change (DASC).

Supplementary Figure 3

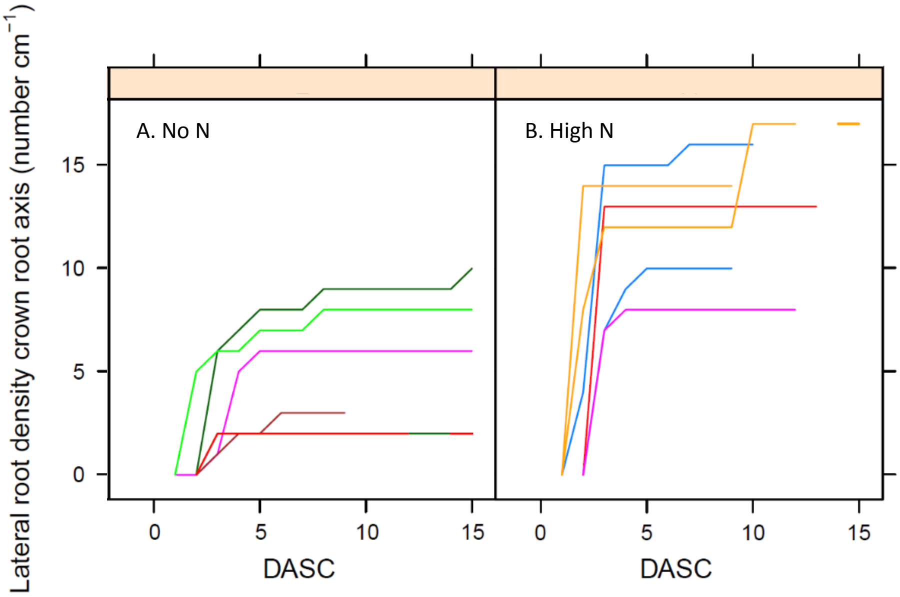

Supplementary Figure 3 Lateral root density on the crown root axis of maize (*Zea mays* L.) for roots grown in a split-root rhizoslide system with half the roots in a no N compartment (A) and the other half in a high N compartment (B) over time. The number in the orange box indicates the plant number. Time is given in days after solution change (DASC).

Supplementary Figure 4

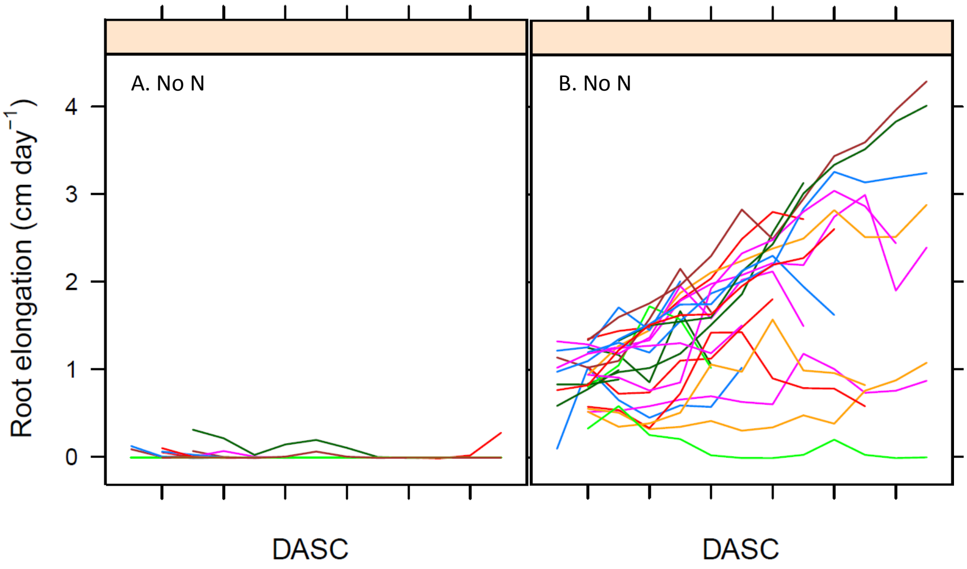

Supplementary Figure 4 Lateral root elongation rates of individual roots of maize (*Zea mays* L.) of crown roots grown in a split-root rhizoslide system with half the roots in a no N compartment (A) and the other half in a high N compartment (B). The number in the orange box indicates the plant number, and different coloured lines indicate different roots. Time is given in days after solution change (DASC).

Supplementary Figure 5

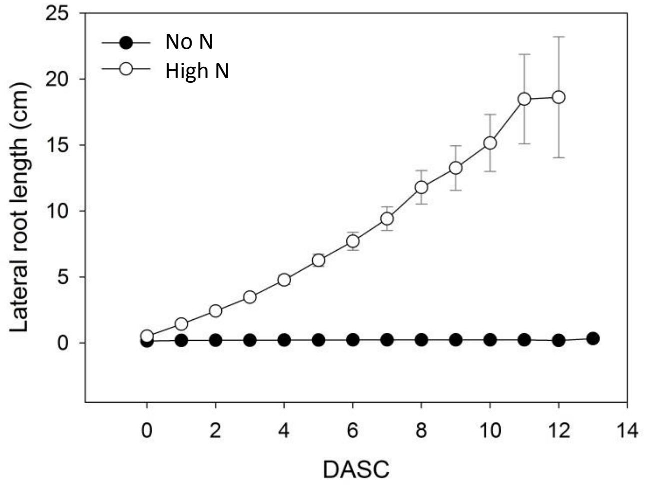

Supplementary Figure 5 Crown lateral root length of *Zea mays* L. plants over time grown in a split-root rhizoslide system with half the root system in no N and the other half in high N. Time is given in days after solution change (DASC).

Supplementary Figure 6

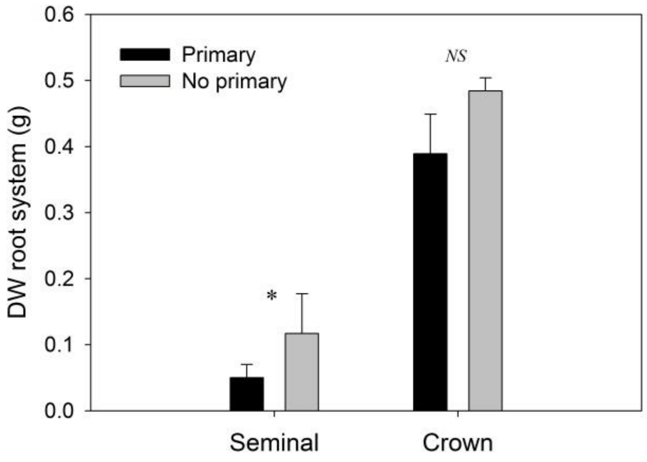

Supplementary Figure 6 Dry weight (DW) of the seminal and crown root system of *Zea mays* plants grown in rhizoslides. The primary root of half the plants was removed 2 d after transplantation into the rhizoslides. Values are means (n=9). Asterisks indicate significant difference between plants with a primary root and plants without a primary root ($p < 0.05$); NS, no significant difference ($p > 0.05$).
